# Effects of Multicomponent Exercise on Functional and Cognitive Parameters of Hypertensive Patients: A Quasi-Experimental Study

**DOI:** 10.1155/2017/1978670

**Published:** 2017-03-19

**Authors:** Hélio José Coelho Junior, Bruno Rodrigues, Daniele Jardim Feriani, Ivan de Oliveira Gonçalves, Ricardo Yukio Asano, Samuel da Silva Aguiar, Marco Carlos Uchida

**Affiliations:** ^1^School of Physical Education, University of Campinas, Campinas, SP, Brazil; ^2^Center of Health Sciences, University of Mogi das Cruzes, Mogi das Cruzes, SP, Brazil; ^3^School of Arts, Sciences and Humanities, University of São Paulo, São Paulo, SP, Brazil; ^4^Brazil School of Physical Education, Catholic University of Brasília, Brasília, DF, Brazil

## Abstract

*Purpose*. The present study aimed to investigate the impact of a 6-month multicomponent exercise program (MCEP) on physical function and cognitive parameters of normotensive (NTS) and hypertensive (HTS) older patients and verify if age can influence the adaptations in response to the exercise.* Methods*. A total of 218 subjects, 101 NTS and 117 HTS, were recruited and underwent functional and cognitive evaluations before and after six months of a MCEP. The program of exercise was performed twice a week, for 26 weeks. The physical exercises were thought to mimic the activities of daily living and, therefore, aggregated functional and walking exercises. Exercise sessions were performed at moderate intensity.* Results*. Data indicated that HTS and NST patients showed a similar increase in the performance of walking speed test and one-leg stand test after the MCEP. Regarding age, results did not show differences in the magnitude of adaptations between old and young HTS and NTS patients.* Conclusions*. Data of the present study indicated that a 6-month MCEP was able to increase equally balance and mobility in NTS and HTS patients. Moreover, data demonstrated that aging did not seem to impair the capacity to adapt in response to exercise in both groups.

## 1. Introduction

The aging process is a continuous phenomenon accompanied by alterations in some physiological systems, collaborating with the development of geriatric syndromes and chronical diseases, such as hypertension (HTN) [1–3]. In fact, HTN has higher prevalence in older people (~70%), which leads to a poor prognosis in this population because patients with high blood pressure show increased risk for stroke and myocardial infarction [4, 5].

However, several evidences have been indicating that HTN not only is strongly associated with cardiovascular risk factors, but also can lead to physical and cognitive impairments in older adults in a time-dependent fashion [6–8]. In turn, aging process per se is already accompanied by marked decrease on physical functioning and on cognitive capacities [9]. Thus, aggregate HTN and aging can potentialize physical and cognitive decline. This phenomenon is a public health problem, once physical functioning and cognitive capacities compose two largest domains: independence and autonomy, respectively, which are analogous to the concept of health in older population [9].

Multicomponent exercise programs (MCEP) emerge as a possible type of physical exercise able to prevent the deleterious effects of aging on the organic system, improving physical functioning, and cognitive capacities [10, 11]. This occurs, since a mix of different exercise regimes are offered in the same exercise routine, thereby not requiring sessions of physical exercise with long duration while developing several physical capacities and skills [11], which can collaborate with exercise adherence in the hypertensive population [12].

Nevertheless, just one study, which was performed with a low-sample size, recorded the effects of a MCEP on functional parameters of hypertensive patients [13]. Moreover, cognitive capacities have not been studied in this context.

Therefore, the present study aimed to investigate the impact of a 6-month MCEP on physical function and cognitive parameters of normotensive (NTS) and hypertensive (HTS) older patients and verify if age can influence the adaptations in response to the exercise.

Based on aforementioned data, the alternative hypothesis* (H1)* of the present study is that MCEP is capable of inducing significant improvements in functional and cognitive parameters of NTS and HTS older adults, regardless of age.

## 2. Materials and Methods

The present study has a quasi-experimental design, which aimed to investigate the effects of a 6-month multicomponent exercise program (MCEP) in the functional and cognitive parameters of normotensive (NTS) and hypertensive (HTS) older patients. Therefore, patients were undergoing functional and cognitive evaluations before and after 6 months of MCEP. All volunteers signed the informed consent form and completed all measurements. This study was approved by the Research Ethics Committee of the University of Campinas (UNICAMP). This study was developed in accordance with the Declaration of Helsinki and according to Resolution 196/96 of the National Health Council.

### 2.1. Subjects

A total of 101 NTS and 117 HTS untrained patients were recruited from two specialized healthcare centers for older adults in a town located in the metropolitan area of São Paulo, Brazil. Volunteers were recruited by convenience and asked verbally by the medical team and researchers about their participation in the study. All subjects provided informed consent before enrolment.

The exclusion criteria were use of hormone replacement and/or psychotropic drugs, cerebrovascular disease (e.g., stroke), pulmonary disease, neurological or psychiatric disease (e.g., Parkinson's or Alzheimer's disease), musculoskeletal disorders, comorbidities associated with greater risk of falls and any kind of dizziness, and blurred vision or lightheadedness when rising or remaining standing for long, which could indicate orthostatic hypertension and/or labyrinthitis. The inclusion criterion was age ≥60 years. Patients of both genders were accepted in the present study. After the application of the exclusion and inclusion criteria, 218 older women were included in the analyses.

The volunteers were subdivided into NTS and HTS groups according to previous clinical diagnosis of HTN. Since both healthcare centers serve a large number of patients and the medical team (i.e., nurse, physician, and physical educator) is of limited size, the pathological conditions were simply recorded by the head physician and head nurse of each center. A specialist (i.e., cardiologist) who was not affiliated to and was outside the center then made the diagnosis of HTN, according to the guidelines [14].

In summary, before the participants began the activities in the centers where they were recruited, a medical consultation was conducted and an extensive list of medical exams was required (e.g., fasting blood glucose, fasting blood insulin). If the patient showed any signal of HTN, such as high blood pressure levels during the first visits in the centers, he/she was invited to measure blood pressure levels, at least, three times, during different periods of the day at home. If her/his blood pressure evaluations remained elevated, he/she was referred to a cardiologist. After they underwent specific medical consultation (i.e., cardiologist) and perform all specific exams (i.e., 24-hour ambulatory blood pressure monitoring [ABPM], home blood pressure) [14], patients should return to the centers with a letter signed by the specialist confirming or not the diagnosis of HTN. It is important to mention that the criteria adopted in HTN diagnosis (i.e., stage 1) proposed by the Brazilian Society of Cardiology (BSC) was based on systolic blood pressure (SBP) values among 140–159 mmHg and/or diastolic blood pressure (DBP) values among 90-90 mmHg [14]. Moreover, repeated measurements in different places were proposed by the cardiologist to confirm the clinical diagnosis of HTN. Final diagnosis was signed by the head physician of the center and a nurse updated the medical records each 6 month. Patients of the present study were recruited one week after the update of the medical records.

All volunteers were instructed to refrain from physical exercise for the 96 hours, as well as drinking coffee, alcoholic, and energy drinks during the 24 hours before all evaluations. Although alimentary ingestion was not controlled, subjects were instructed to maintain the habitual food intake throughout the period of the protocol. All volunteers ensure that these parameters were not altered during the protocol. Evaluations (pre and post) were performed 120 hours before and after the beginning and the end of the physical exercise program, respectively.

### 2.2. Functional Evaluations

Before the performance of the tests, an experienced researcher detailed the procedures of each test. The volunteers performed all tests twice and the higher value recorded in each test was used in the analysis. During all tests, verbal encouragement was provided to ensure that volunteers reached the best performance possible. The present protocol was used by our group elsewhere [42].

#### 2.2.1. Sit-to-Stand Test

Volunteers were requested to rise from a chair five times as fast as possible with their arms crossed in front of the body. The stopwatch was activated when the volunteer raised their buttocks off the chair and was stopped when the volunteer seated back.

#### 2.2.2. One-Leg Stand Test

The one-leg stand test was performed with the volunteers standing in a unipodal stance with the dominant lower limb, the contralateral knee remaining flexed at 90°, the arms remaining crossed in front of the chest, and the head being straight. A stopwatch (Moure Jar®, China) was activated when the volunteer raised their contralateral foot off the floor and was stopped when the contralateral foot touched the floor again.

#### 2.2.3. Usual and Maximal Walking Speed Test

To measure walking speed, a three-meter walking speed test was performed. Volunteers were required to walk a distance of five meters at their usual and fastest possible cadences (without running). Before the evaluation, both feet of each volunteer were to remain on the starting line. Measurement was initiated when a foot reached the one-meter line and was stopped when a foot reached the four-meter line. The one-meter intervals at the beginning and end were used to avoid early acceleration and/or deceleration.

#### 2.2.4. Time Up and Go (TUG) Test

The Time Up and Go (TUG) test involved getting up from a chair without the help of the arms, walking a distance of three meters around a marker placed on the floor, coming back to the same position, and sitting back on the chair. The test began when a researcher shouted a “go!” command. The stopwatch was activated when the volunteers got up from the chair and was stopped when they were seated again.

### 2.3. Cognitive Evaluation-Executive Function (EF)

#### 2.3.1. TUG Cognitive Test

TUG cognitive test was accomplished to evaluate EF. This test is performed equally to the conventional TUG; however, a cognitive task (verbal fluency, animal category) must be accomplished during the motor task. Therefore, after the signal of the evaluator, the volunteer performed the route—stand up from the chair, walk three meters, turn around, walk three meters back, and sit down again—naming as many animals as he/she could remember. This task was performed out loud, allowing the evaluators to confirm if the volunteers were, in fact, accomplishing the task. The time spent to perform the task was recorded for evaluation [15].

### 2.4. Multicomponent Exercise Program (MCEP)

The MCEP was performed twice a week, on nonconsecutive days, during 26 weeks at the fitness center of an institutional center for elderly care and living (Centro de Convivência do Idoso [CCI]), Poá, Brazil. The program was designed to offer exercises that would mimic activities of daily live (ADL) gestures, thereby inducing neuromuscular adaptations to maintain the subjects able to perform the ADL. Each exercise session was composed of twelve different exercises stations. The structure of each exercise session was defined by the sequence of one functional exercise followed, immediately, by a brief walk. Therefore, exercise session was composed of approximately 12 minutes of functional exercises, 24 minutes of walk, and 12 minutes of rest. Approximately each session of exercise was composed of 50 patients. A physical trainer professional with larger expertise on exercise training to older people supervised all sessions (OI). Volunteers were instructed to avoid the Valsalva maneuver during the performance of exercises.

The functional exercises were changed during the whole program. However, they always represented ADL with a large necessity of the activity of the lower limbs, as, for example, standing and sitting on the chair, picking up a weight off the floor and putting it on top of a structure, and transferring a weight from one place to another. Balance and proprioception exercises were also composed of functional exercises, as one-leg stand. At most three balance and/or proprioception exercises were used in each session. To complete the list of physical exercises, upper limbs resistance exercises were added.

All functional exercises were performed for one minute. The brief walk was performed for two minutes. Thus, after the end of each functional exercise, volunteers must walk from one point to another (30 m), around a cone, come back to the initial line (30 m), and start the path again until completing the two minutes. A rest interval of 60 seconds was adopted between the stations.

### 2.5. Exercise Intensity Control

The control of exercise intensity was accomplished by the rating of perceived exertion (RPE) method using the adapted Borg scale (2001) (i.e., CR-10) [16], which was used to ensure that volunteers performed the exercises in the aimed intensity. This scale is composed by eleven numbers (i.e., 0, 1, 2, 3, 4, and so on) and eight descriptors (i.e., rest; very, very easy; easy; moderate; somewhat hard; hard; very hard; and maximal), which represents the perception of effort of the subject in front of an exercise load. The higher the reported number, the greater the sensation of effort [16]. During the performance of functional—except for balance exercises—and resistance exercises, volunteers were instructed to maintain the physical activity intensity in 3–5—which represents moderate (i.e., 3), somewhat hard (i.e., 4), and hard (i.e., 5) descriptors. To that, a large picture of RPE scale (i.e., 4 meters high and 1.30 meters wide) was positioned on the wall in the gym's room. The increase in the exercise intensity was based on alterations in the cadence of the performance (i.e., faster), for functional exercises and walk. Moreover, for resistance exercises, volunteers could use elastic bands (EXTEX Sports, São Paulo, Brazil) and dumbbells to reach the intensity prescribed.

*Determination of the Prevalence of Pathologies and Use of Medications*. The prevalence of pathologies and consumption of medications was determined by reviewing the medical records of each subject. As aforementioned, the head physician and the head nurse of the medical centers recorded the pathological conditions, and a specialist not affiliated with the centers made the pathologic diagnoses. Records were reviewed by two researchers (HJCJ and SSA), and data were compared.

### 2.6. Statistical Analyses

Normality of data was tested using the* Kolmogorov-Smirnov* test. Since data did not demonstrate normal distribution, nonparametric statistics were performed. Baseline comparisons between the groups for age and morphological (i.e., body mass, height, and body mass index [BMI]) and hemodynamic (i.e., systolic blood pressure [SBP], diastolic blood pressure [DBP], and mean arterial pressure [MAP]) parameters were performed using two-sided Mann–Whitney test for unpaired samples. Intragroup comparisons in the different periods* (pre, post)* were performed using Wilcoxon matched-pairs. Kruskal-Wallis test was performed to compare the differences of intergroup (mean and delta [%  Δ]). Cohen's effect size (ES) *d* was calculated to assess the magnitude of the results. Cohen's effect size *d* was calculated to assess the magnitude of the results. The effect size was classified according to Rhea, 2004 [17] for untrained volunteers [17]. The level of significance was 5% (*P* < 0.05) and all procedures were performed using the GraphPad Prism 6.0. (San Diego, California, USA).

## 3. Results

Subjects did not show adverse events during exercise sessions and evaluations, and they were not absent for more than three exercise sessions. There were no dropouts during the study, so that the adherence to the physical exercise program was 100%. Volunteers did not report any changes in food intake and in the number or class of medications use during the whole course of the present study.

Table 1 shows the comparison between the groups regarding morphological and hemodynamic parameters. Data demonstrated that HTS patients were older and heavier and had higher BMI, SBP, DBP, and MAP than NTS patients.

Table 2 shows the effects of multicomponent physical exercise on functional and cognitive parameters, as well as the ES, in NTS and HTS patients. One-leg stand and usual and maximal walking speed tests showed significant increase after exercise (pre-post) in both groups. ES classification was* small *for one-leg stand test and* trivial* for usual and maximal walking speed tests in NTS. Similarly, alterations on one-leg stand and usual walking speed tests were classified as* trivial*, whereas maximal walking speed test received* small* classification in HTS. Therefore, ES results indicate that improvements in functional parameters were similar in NTS and HTS.

On the other hand, sit-to-stand, TUG, and TUG with a cognitive task did not show significant alterations. Qualitative analyses are in concordance with the hypothesis test, once the ES for sit-to-stand, TUG, and TUG with a cognitive task were near zero in NTS and HTS patients.

Table 3 shows the comparison between the magnitudes of alterations (%  Δ) on functional and cognitive tests after multicomponent physical exercise in NTS and HTS patients. The results are congruent with the ES shown in Table 1, since there were no significant differences among the groups in relation to the magnitude of alterations after the MCEP.

The effects of multicomponent physical exercise on functional and cognitive parameters of NTS and HTS patients adjusted by age (<75 and ≥75) are shown in Table 4. All groups, except NTS ≥75, showed significant increase in the performance of one-leg stand and on maximal walking speed tests. In relation to one-leg stand, ES classification was* small *for all groups that showed a significant increase after MCEP (i.e., NTS < 75, HTS < 75, and HTS ≥ 75), whereas the alterations shown in NTS ≥75 were just classified as* trivial*, confirming the results observed in the hypothesis test. Interestingly, ES classification of maximal walking speed test was not in concordance with the hypothesis test. In fact, alterations after MCEP on NTS <75 were classified as* moderate*. On the other hand, alterations on HTS <75 and HTS ≥75—which demonstrated significant increase in one-leg stand performance—and on NTS ≥75—which did not demonstrate significant alterations—were classified as* trivial*.

In turn, time to perform usual walking speed test was decreased in the youngest groups (i.e., NTS < 75 and HTS < 75), but not in the oldest groups (i.e., NTS ≥ 75 and HTS ≥ 75). Similar with the results from the maximal walking speed test, ES classification was not congruent with the hypothesis test, and ES classification was* small* for HTS <75 and* trivial* for NTS < 75, NTS ≥ 75, and HTS ≥ 75. Taken together, ES classifications in the usual and maximal walking speed tests could indicate that young groups showed a larger magnitude of alterations after the MCEP in comparison with the oldest groups.

The comparison between the magnitudes of alterations (%  Δ) after multicomponent physical exercise in NTS and HTS patients adjusted by age is shown in the Table 5. Data refuted the hypothesis created by ES classification and did not indicate significant differences between the groups.

## 4. Discussion

The present study aimed to investigate and compare the effects of 6 months of a MCEP on the functional and cognitive parameters of NTS and HTS patients. Furthermore, we wished to verify if age was a confounding factor in this phenomenon, affecting the capacity of HTS patients to adapt to physical stimulus.

We hypothesized that the MCEP would be able to improve functional and cognitive parameters of NTS and HTS patients, regardless of age. Nevertheless, data of the present study partially refuted our* H1*, since improved functional capacity was observed (i.e., balance and mobility) in both, NTS and HTS patients, after the MCEP. However, cognitive capacities were not altered in any of the groups. In addition, when a new set of analyses were performed with the subgroups of the NTS and HTS, <75 and ≥75, results did not demonstrate decreased functional adaptations on HTS ≥75 subgroup. However, significant results observed in NTS groups (Table 2) were reversed in NTS ≥75.

Regarding functional capacities (Tables 2 and 3), NTS and HTS patients increased similarly the time in the balance test (i.e., one-leg stand), 73% and 88%, concomitantly with decreased time spent to perform usual walking speed, −9.8% and −7.8%, and maximal walking speed tests, −32.4% and −36.5%, respectively, after 6 months of MCEP. Several evidences in the literature have confirmed our data, demonstrating improvements in balance and mobility in NTS patients after MCEP, even after short-term exercise programs (e.g., 4 weeks) and in frailty older adults [18–20].

However, despite these beneficial results of MCEP in NTS patients, just one study [13] investigated the effects of MCEP on a sample composed exclusively by hypertensive patients. In this experiment, de Moraes et al. [13] made 32 HTS patients undergo 60 minutes of MCEP, two days per week, for 3 months. Similar to the present study, the sessions of exercise were composed by walk and resistance exercises, at moderate to vigorous intensity. However, authors added dance exercises. Results demonstrated that MCEP was able to elicit significant improvement in lower-*limb muscle strength*, balance, and mobility [13].

Even if the present study also observed improvements in balance and mobility after MCEP, indicated by one-leg stand and walking speed, respectively, increase in lower-limb muscle strength was not shown by NTS and HTS patients. Interestingly, the time of intervention in the experiment performed by de Moraes et al. [13] was shorter than in the present study (3 months versus 6 months). An interference of pathological condition (i.e., hypertension) is unlikely, since results were not observed in NTS patients. An initial hypothesis to explain different outcomes among the studies could be differences on baseline physical levels between the samples. However, scores on sit-to-stand test—performed in both groups for lower-limb muscle strength evaluation—were similar in both studies, even after division of the groups by age.

In turn, the dissimilarities among the protocols of MCEP can be indicated as a possible factor responsible for different results observed in the comparison. In fact, muscle strength is composed basically of a morphological (i.e., muscle mass) and a neural component (i.e., muscle recruitment) [21, 22]. During aging, several mechanisms (e.g., hormonal, neural, and immunological) lead to marked decrease in muscle mass, muscle atrophy, and muscle strength—dynapenia—collaborating to development of sarcopenic phenotype, which is closely associated with poor prognosis (e.g., increase in sedentary behavior, low functionality, risk of falls, and diseases prevalence) [1, 23]. Maintenance or improvement of muscle strength during aging in response to physical exercise is generally associated with increase in muscle mass [24–26], but not exclusively, once evidences demonstrate increase in muscle strength even in the absence of apparent muscular hypertrophy [27, 28], which occur predominately in response to neuromuscular adaptations [25–28]. Yet, both factors can occur in response to physical exercise and collaborate to increase in muscle strength [25, 26]. Moreover, some authors suggest that the increase in muscle strength after programs of physical exercise is dependent on muscular tension generated during muscular contractions, which is associated with external load (e.g., intensity, during muscle contraction) [24].

In the present study, each session of MCEP (~48 minutes) was composed predominantly by an aerobic component, due the time of walk performed between functional exercises (~24 minutes [50%]) and the time of walk performed inside the functional exercises, since some exercises required that the volunteer translocase from one location to another for one minute. This characteristic is a possible explanation for the lack of changes in muscle strength in the present study, since aerobic stimulus requires the predominant action of slow-twitch muscle fibers (type I), which have a high capacity to keep muscle working for a long time, avoiding muscular fatigue, considering that the resistance imposed to skeletal muscle contraction occurs in a low and/or low to moderate levels. However, these fibers have low capacity to generate tension, which impairs their capacity to cause large improvement in muscle strength and muscle mass. Indeed, evidences indicate that older adults who engaged for a long time in physical exercise programs with high aerobic component show muscle strength and muscle mass values similar to sedentary matched-controls [29].

Furthermore, both studies, the present study and the study of de Moraes et al. [13], showed different relative time of functional exercises on MCEP, 25% and 33.3%, respectively. Yet, the present protocol of MCEP was composed of just a single set of each functional exercise, which was characterized predominantly by resistance exercises (66,6%) and also by balance and proprioceptive exercises (33,3%). Regarding resistance exercises, which is more associated with muscle mass and muscle strength development than balance and proprioceptive exercises, several evidences from original studies, corroborated by meta-analytic regressions, have been indicating that programs composed of single sets, even at high intensity, are limited to elicit improvement in muscle mass and strength, including in older adults [30–32].

Therefore, taken together, these data indicate that, due to the prevalence of aerobic components in the exercise session, added to an inappropriate resistance component, the protocol of MCEP used in the present study was not a profitable stimulus to elicit morphological changes in muscular architecture, as well as being able to reach a threshold necessary to cause adaptations of the neuromuscular components associated with improvement in muscle strength. However, interestingly, the lack of changes in muscle strength did not impair the improvements in the performance of usual and maximal walking speed, as well as on balance test, observed in both groups after MCEP.

In this sense, it is known that muscle power, compared with muscle strength, seems to decrease early and in a larger magnitude during aging [33, 34] and it seems to be the main functional limitation predictor in older adults [35]. Several evidences have been demonstrating that muscle power can be more associated with functional and physical capacities in older adults, including walking speed and balance, than muscle strength [33, 36]. Moreover, increase in muscle power has been observed after programs of exercise with different intensities (e.g., low, moderate, and high), since this kind of adaptation is closely associated with the velocity of muscle contraction, and muscle tension is not a determinant factor to induce its alterations [37–39]. Thus, it is possible to infer that patients of the present study could show increase in muscle power, even without changes in muscle strength, after 6 months of MCEP. However, more studies assessing this capacity are necessary to confirm our hypothesis.

Besides the functional tests, the present study also investigated the effects of MCEP on EF in NTS and HTS patients, through dual task (TUG associated with a cognitive task). EF is a cognitive capacity composed of other cognitive domains (e.g., shifting, working memory, and inhibition), which allow the subject to create, develop, and perform a strategy to perform an aim, evaluate the outcomes, and, if necessary, change the strategy during or at the end of the task, creating new strategies for the future [40]. Therefore, EF is an essential cognitive domain for maintenance of autonomy and independence during aging, and its impairment is observed in intermediate conditions (e.g., mild cognitive impairment), as well as during several psychopathological conditions, including Alzheimer's disease [15].

Just few evidences evaluated the effect of MCEP on cognitive domains in older adults [20, 41]. However, protocols investigated different samples (i.e., frailty) and in the study developed by Tarazona-Santabalbina et al. [20], volunteers also underwent nutritional supplementation, which can impair comparisons and inferences. Different from the present study, Eggenberger et al. [41] observed significant increase in EF after 6 months of MCEP. The cognitive regulatory mechanisms behind the modulation of physical exercise variables are still unclear and limit our inferences. However, different volumes of MCEP (48 minutes versus 60 minutes) and exercise resistance component are plausible explanations. Yet, different evaluation tools can help to explain dissimilarities, since EF in the aforementioned study was evaluated by a questionnaire test (i.e., Trail Making Test, Part B), while the present study added a verbal fluency test to a motor test. Therefore, more studies are still necessary.

This study was also developed to verify if age plus pathological condition (i.e., hypertension) could be a confounding factor in the magnitude of adaptations after MCEP. Our data accepted null hypothesis and show that age did not seem to alter the response of HTS patients after MCEP. Cross-sectional studies have been discussing the possibility that HTS patients present impairment on physical function [7, 8, 42]. However, results have been shown to be controversial.

Plausibility besides its theory is based on the continuous vascular damage in the arteries responsible for the transport of blood to the brain areas accountable for mobility (e.g., motor cortex), which could impair muscle movement throughout time and, consequently, muscle adaptations in response to physical exercise [7]. Moreover, evidences suggest that hypertensive patients could present a higher prevalence and magnitude of progression of white matter hyperintensities (WMHs) in comparison with normotensive patients [6, 43]. Yet, this phenomenon seems to be associated with decline in physical functioning [6]. Therefore, data of the present study (Tables 4 and 5) added evidences to cross-sectional studies and, for the first time, demonstrated that aged HTS patients did not present impairment in the capacity of adaptation in response to physical stimulus.

However, it is important to mention that a Mann–Whitney test was performed between HTS < 75 and HTS > 75 groups to evaluate differences between functional and cognitive tests. Again, data did not demonstrate significant differences between the magnitudes of alterations. Nonetheless, the *P* value for balance test was equal to 0.05. Interestingly, in the seminal study from Hausdorff et al. [8], the authors observed lower performance in the balance test in hypertensive patients being ~75 years old. Moreover, an association among high blood pressure values and poor balance performance was recorded [8]. Rosano et al. [6] showed an age-dependent association between WMHs, well-functioning hypertensive patients, and physical declining [6]. Therefore, it is possible that the results of the present study are not observed in patients with secondary morbidities (e.g., stroke) and older patients and in response to other kinds of physical exercise. Future studies should explore these factors.

On the other hand, NTS ≥ 75 patients did not present significant improvement in performance after MCEP. These data are complicated to be discussed, since scientific literature pointed out the possibility that HTS patients show impairment in adaptations, but not NTS. In our view, the sample size (*n* = 5) of the present study is a limitation factor and probably responsible for this absence of alterations. Thus, we encourage future studies with larger samples of NTS ≥ 75.

Other potential limitations of our study should be mentioned. The present study has a quasi-experimental design. Therefore, inherent characteristics of this kind of approach, as the lack of randomization and the absence of a control group (sedentary NTS and HTS), must be assumed. Moreover, future studies that aim to use MCEP should increase the number of sets of resistance training in the protocol, since this approach can collaborate to increase in the magnitude of alterations after MCEP, mainly in muscle strength. Due to all the aforementioned data, which highlight the importance of muscle power in the context of aging and functional capacity, the measurement of dynamic muscle power should be added in the methodology. However, the present study indicates that this model of MCEP should be studied in better design models of scientific studies (i.e., clinical randomized trial). Lastly, more information about the cognitive status and level of education of the volunteers of the present study was not collected. These data are important and could collaborate to a better explanation about the results shown in the present study.

Therefore, the present study indicates that a 6-month MCEP is able to increase equally balance (i.e., one-leg stand test) and mobility (i.e., usual and maximal walking speed test) in NTS and HTN adults. Therefore, aging did not seem to be a confounding factor, impairing the capacity of older HTS patients to adapt to physical stimulus. Thus, the current data indicate that this MCEP can be used to improve muscle functionality of several hypertensive patients, independently of age. This seems to be a good option to public health programs that have the necessity to offer short-time services to several patients in the same time.

## Figures and Tables

**Table 1:** Comparison between the groups regarding the morphological and hemodynamic parameters.

Variables	NTS (*n* = 101)	HTS (*n* = 117)
Subjects characteristics		
Age (years)	64.1 ± 6.8	66.3 ± 6.9^*∗*^
Body mass (kg)	65.0 ± 12.1	71.5 ± 13.3^*∗*^
Height (cm)	156.3 ± 0.1	158.4 ± 0.6
Body mass index (kg/m^2^)	26.1 ± 5.0	28.5 ± 4.8^*∗*^
Hemodynamic parameters		
SBP (mmHg)	124.9 ± 16.2	135.2 ± 18.1^*∗*^
DBP (mmHg)	72.9 ± 11.1	79.2 ± 10.4^*∗*^
MAP (mmHg)	89.8 ± 17.0	96.2 ± 17.0^*∗*^

Data are presented as mean ± SD. NTS = normotensive group; HTS = hypertensive group; SBP = systolic blood pressure; DBP = diastolic blood pressure; MAP = mean arterial pressure.

^*∗*^ *P* < 0.05 versus NTS.

**Table 2:** Effect size and its classification of behavior of functional parameters after the experimental session.

Variable		NTS	HTS
Sit-to-stand (repetitions)	Pre	10.8 ± 2.8	10.4 ± 2.1
	Post	10.6 ± 2.7	10.2 ± 2.8
	ES	0.07 (trivial)	0.08 (trivial)

One-leg stand (s)	Pre	17.6 ± 13.2	14.9 ± 12.3
	Post	25.6 ± 7.5^*∗*^	22.5 ± 9.1^*∗*^
	ES	−0.74 (small)	0.15 (trivial)

Usual walking speed (m/s)	Pre	0.88 ± 0.62	0.83 ± 0.24
	Post	0.70 ± 0.11^*∗*^	0.73 ± 0.19^*∗*^
	ES	0.40 (trivial)	0.46 (trivial)

Maximal walking speed (m/s)	Pre	1.06 ± 1.26	0.94 ± 0.55
	Post	0.55 ± 0.8^*∗*^	0.56 ± 0.12^*∗*^
	ES	0.48 (trivial)	0.95 (small)

TUG (s)	Pre	6.5 ± 1.3	6.9 ± 1.1
	Post	6.6 ± 1.2	6.8 ± 1.6
	ES	−0.07 (trivial)	0.07 (trivial)

TUG with a cognitive task (s)	Pre	7.5 ± 2.1	7.8 ± 1.8
	Post	7.6 ± 1.8	8.1 ± 2.2
	ES	−0.05 (trivial)	−0.14 (trivial)

^*∗*^ *P* < 0.05 versus pre; NTS = normotensive; HTS = hypertensive; TUG = Time Up and Go; ES = effect size.

**Table 3:** Magnitude (%  Δ) of effect in both groups.

Variable		NTS	HTS
One-leg stand (s)	% Δ	45.4	51.0
Usual walking speed (m/s)	% Δ	−20.4	−12.0
Maximal walking speed (m/s)	% Δ	−48.1	−40.4
Sit-to-stand (repetitions)	% Δ	−1.5	−1.9
TUG (s)	% Δ	1.5	−1.4
TUG with a cognitive task (s)	% Δ	1.3	3.8

NTS = normotensive; HTS = hypertensive; TUG = Time Up and Go.

**Table 4:** Effect size and its classification of behavior of functional parameters after the experimental session adjusted by age.

Variable		NTS < 75 (*n* = 96)	NTS ≥ 75 (*n* = 5)	HTS < 75 (*n* = 102)	HTS ≥ 75 (*n* = 15)
Sit-to-stand (repetitions)	Pre	10.7 ± 2.8	10.8 ± 1.0	10.2 ± 2.0	11.7 ± 2.5
	Post	10.5 ± 2.7	12.8 ± 1.5	9.9 ± 2.0	12.1 ± 2.7
	ES	0.07 (trivial)	−1.56 (moderate)	0.14 (trivial)	−0.15 (trivial)

One-leg stand (s)	Pre	17.5 ± 13.3	18.5 ± 10.3	15.8 ± 2.5	7.5 ± 6.0
	Post	26.2 ± 7.0^*∗*^	15.7 ± 2.6	22.8 ± 9.2^*∗*^	18.3 ± 9.6^*∗*^
	ES	−0.81 (small)	0.37 (trivial)	−1.03 (small)	−1.34 (small)

Usual walking speed (m/s)	Pre	0.88 ± 0.64	0.80 ± 0.18	0.83 ± 0.24	0.89 ± 0.30
	Post	0.69 ± 0.11^*∗*^	0.77 ± 0.18	0.71 ± 0.18^*∗*^	0.85 ± 0.19
	ES	0.41 (trivial)	0.16 (trivial)	0.56 (small)	0.15 (trivial)

Maximal walking speed (m/s)	Pre	0.89 ± 0.36	0.92 ± 0.38	1.06 ± 1.24	0.93 ± 0.39
	Post	0.54 ± 0.08^*∗*^	0.57 ± 0.11	0.54 ± 0.11^*∗*^	0.62 ± 0.11^*∗*^
	ES	1.34 (moderate)	1.25 (small)	0.59 (small)	1.08 (small)

TUG (s)	Pre	6.5 ± 1.3	6.6 ± 1.0	6.7 ± 1.0	8.0 ± 1.4
	Post	6.6 ± 1.3	6.8 ± 0.8	6.6 ± 1.4	7.6 ± 1.9
	ES	−0.07 (trivial)	−0.22 (trivial)	0.08 (trivial)	1.19 (moderate)

TUG with a cognitive task (s)	Pre	7.5 ± 2.1	7.9 ± 1.0	7.6 ± 1.7	9.2 ± 1.8
	Post	7.5 ± 1.8	9.2 ± 1.6	7.9 ± 2.1	9.5 ± 2.2
	ES	0 (trivial)	−0.97 (small)	−0.15 (trivial)	−0.14 (trivial)

^*∗*^ *P* < 0.05 versus pre; NTS = normotensive; HTS = hypertensive; TUG = Time Up and Go; ES = effect size.

**Table 5:** Magnitude (%  Δ) of effect in both groups adjusted by age.

Variable		NTS < 75	NTS ≥ 75	HTS < 75	HTS ≥ 75
One-leg stand (s)	% Δ	80.7	29.3	182.9	179.9
Usual walking speed (m/s)	% Δ	−9.9	−3.6	−9.0	−5.1
Maximal walking speed (m/s)	% Δ	−32.16	−32.9	−35.2	−30.4
Sit-to-stand (repetitions)	% Δ	3.7	19.3	2.6	7.7
TUG (s)	% Δ	−3.4	−3.3	−1.3	2.5
TUG with a cognitive task (s)	% Δ	6.3	17.3	7.2	8.4

NTS = normotensive; HTS = hypertensive; TUG = Time Up and Go.
